# Emerging Chemical, Biochemical, and Non-Thermal Physical Treatments in the Production of Hypoallergenic Plant Protein Ingredients

**DOI:** 10.3390/foods13142180

**Published:** 2024-07-11

**Authors:** Joan Oñate Narciso, Saqib Gulzar, Robert Soliva-Fortuny, Olga Martín-Belloso

**Affiliations:** 1 Department of Food Technology, Engineering and Science, University of Lleida, Av. Alcalde Rovira Roure 191, 25198 Lleida, Spain; saqib.gulzar@udl.cat (S.G.); robert.soliva@udl.cat (R.S.-F.); olga.martin@udl.cat (O.M.-B.); 2 Agrotecnio Center, Av. Alcalde Rovira Roure 191, 25198 Lleida, Spain

**Keywords:** glycation, polyphenol complexation, protein–polysaccharide complexation, fermentation, enzymatic catalysis, food allergen

## Abstract

Allergies towards gluten and legumes (such as, soybean, peanut, and faba bean) are a global issue and, occasionally, can be fatal. At the same time, an increasing number of households are shifting to plant protein ingredients from these sources, which application and consumption are limited by said food allergies. Children, the elderly, and people with immune diseases are particularly at risk when consuming these plant proteins. Finding ways to reduce or eliminate the allergenicity of gluten, soybean, peanut, and faba bean is becoming crucial. While thermal and pH treatments are often not sufficient, chemical processes such as glycation, polyphenol conjugation, and polysaccharide complexation, as well as controlled biochemical approaches, such as fermentation and enzyme catalysis, are more successful. Non-thermal treatments such as microwave, high pressure, and ultrasonication can be used prior to further chemical and/or biochemical processing. This paper presents an up-to-date review of promising chemical, biochemical, and non-thermal physical treatments that can be used in the food industry to reduce or eliminate food allergenicity.

## 1. Introduction

Over the past few years, an increasing number of consumers have shifted to plant-based protein ingredients for environmental, health, and animal welfare reasons. Among children, the adoption of meat analogs and plant-based proteins is spurred by their empathy towards animals, contributing to their positive perception of these alternatives [1]; meanwhile, adults with weight and health concerns are more likely to buy plant-based foods/alternatives due to their health benefits [2]. For instance, the consumption of nuts and legumes such as peas, faba beans, lentils, chickpeas, and peanuts has been linked to lower coronary heart disease incidence and a decrease in total and low-density lipoprotein cholesterol [3,4]. Despite their positive health effects, some of these ingredients cause allergenic or immunologic reactions when consumed. Wheat gluten, which is mainly composed of gliadins and glutenins, can trigger immune reactions in people suffering from celiac disease, while peas, faba beans, lentils, chickpeas, and peanuts contain the cupin and prolamin superfamilies of food allergens [5]. It is important to address gluten and legume food allergenicity in the general population, with special care towards children, the elderly, and immunocompromised people. The abovementioned legumes and gluten proteins are highlighted in this study because they are common plant protein ingredients known to elicit allergenic reactions.

**Figure 1:**
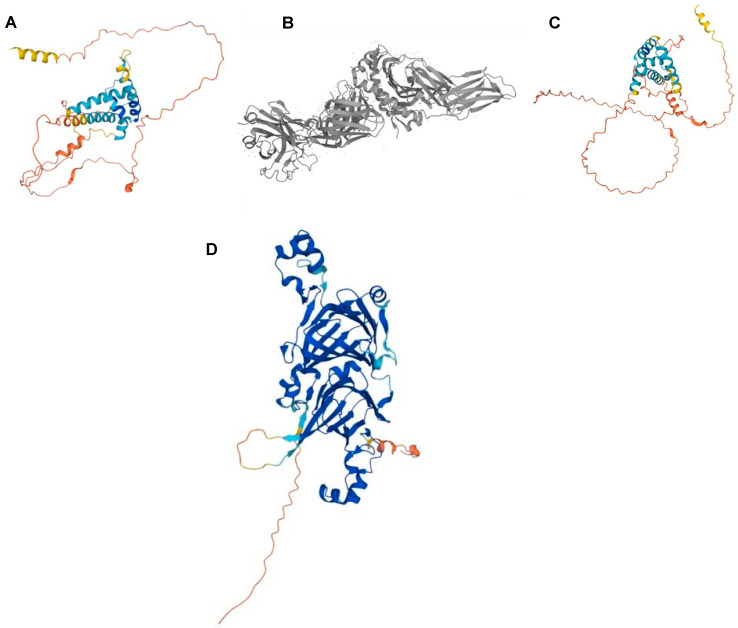
Protein structures and their conformations can suggest food allergen stability. The content of β-sheet is positively correlated with the stability of the secondary structure of a protein. Gluten structures from wheat (*Triticum aestivum*): (**A**) low-molecular-weight glutenin subunit 1D1 (accession: P10386); (**B**) α/β-gliadin MM1 from amino acid positions 246–260 (accession: P18573); (**C**) γ-gliadin (accession: P21292); and (**D**) β-Conglycinin beta subunit 2 (Gly m 5) from soybean (*Glycine max*) showing the “jellyroll” (double-stranded β-helix) structure that contributes to its thermal stability (accession: F7J077; taken from UniProt).

Three protein families introduced in this review have been previously implicated in food allergenicity and sensitivity—namely, glutens, cupins, and prolamins. The first is a common cause of food sensitivity. This is because, among the various gluten proteins, gliadin and glutenin are rich in glutamine (38%) and proline (20%) [6], and specifically, low-molecular-weight glutenin, α-gliadin, and γ-gliadin have the highest IgE reactivities [5] (Figure 1A–C). Indeed, the high proline and glutamine content of gliadin makes it difficult to digest for proteases in the gastrointestinal tract, thus allowing it to possibly escape the human gut undigested, triggering immune reactions in celiac disease [7]. It is also heat-stable [7], making gluten processing through thermal treatments alone not sufficient to reduce its allergenicity.

Some food allergens belong to cupins, a large protein superfamily possessing a common structure described as a “double-stranded β-helix” or a barrel-like “jellyroll” (Figure 1D). Among these are the 7S globulins of soybeans (β-conglycinin) and peanuts (conarachin; Ara h 1) and their 11S globulin counterparts, glycinin and arachin (Ara h 3), respectively [8]. Cupins are generally very stable due to the thermal denaturation- and proteolysis-resistant double-stranded β-helix, resulting in immunologically active fragments that survive in the gastrointestinal tract.

**Figure 2:**
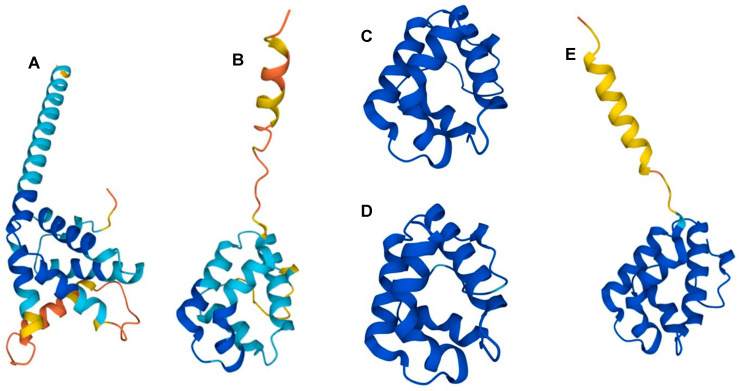
Structures of proteins from the prolamin superfamilies showing similarities and potential cross-reactivities: (**A**) 2S seed storage albumin protein from soybeans (*Glycine max*) (accession: P19594); (**B**) Ara h 9 allergen from peanuts (*Arachis hypogaea*) (accession: B6CEX8); (**C**) Pru p 3 allergen from peaches (*Prunus persica*) (accession: P81402); (**D**) Pru ar 3 allergen from apricots (*Prunus armeniaca*) (accession: P81651); and (**E**) Mal d 3 allergen from apples (*Malus domestica*) (accession: Q9M5X7) (all taken from UniProt).

Some food allergens can be classified under the prolamin superfamily, such as the 2S albumin seed storage and nonspecific lipid transfer proteins (nsLTPs). The peanut allergens Ara h 2, 6, and 7 are related to the former [9], while the Ara h 9 allergen—seemingly the most reactive to people from the Mediterranean region—belongs to the latter [10,11] (Figure 2A,B). Interestingly, nsLTPs from other plant species, such as peach (Pru p 3) [12,13], apple (Mal d 3) [14], and apricot (Pru ar 3) [15], are also reactive allergens among Mediterranean populations (Figure 2C–E). The nsLTPs are resistant to many different treatments and conditions, such as proteolysis, a harsh pH, or heat, and can also refold into their native structure upon cooling [16].

Because of the resistance of these protein allergens to gastric digestion and their particular stability in heat and harsh pH conditions, other means of modifying their structures and hindering their antigenic epitopes from eliciting allergic reactions should be devised. Chemical reactions such as glycation and polyphenol conjugation, as well as enzymatic/microbial fermentation and catalysis (degradation and/or cross-linking by enzymes), are promising ways to break down/mask the antigenic epitopes and destroy the stability of these allergens. The effectiveness of these reactions can be enhanced using physical treatments—namely, microwaves, high pressure, or ultrasound—prior to chemical/biochemical ones. Another novel strategy is protein–polysaccharide complexation for liquid media such as soymilk. This review includes a compilation of studies, published in the past fifteen years, focusing on allergenicity reduction in gluten, soybean, peanut, and faba bean proteins using the processes mentioned previously and discusses the future of allergen-free foods from the perspective of these processing techniques.

## 2. Chemical Processes to Reduce Food Allergenicity

### 2.1. Glycation and Glycosylation

Protein allergenicity is related to allergen epitopes, particularly their amino acid sequence and, subsequently, their conformation. Usually, when the epitopes of a protein antigen or allergen attach or bind to our antibodies, such as immunoglobulin E (IgE), allergenic reactions start. Changes in the binding epitope conformation by denaturation, for example, can lead to a reduction in the ability of the allergens to bind to IgE, reducing their allergenicity [17]. Similar effects can be induced by protein glycation and glycosylation. The former, for example, may result in epitope conformation masking or destruction through the formation of protein–sugar aggregates, potentially reducing allergenicity [18]. Although glycation and glycosylation processes may appear similar, they follow different mechanisms: the former is a non-enzymatic glycosylation (also called the Maillard reaction or the amino–carbonyl reaction), while the latter is a glycosyltransferase-catalyzed enzymatic reaction. 

#### 2.1.1. Gluten Proteins

The effects of glycation on gluten allergenicity remain largely unexplored. In [19], when gluten was conjugated with maltose via a Maillard reaction under dry-heated conditions, a β-fold and β-turn decrease and an α-helix increase were observed at a certain degree, with little effect on the irregularly coiled structure of gluten after the reaction. In another study, the conjugation of gluten with konjac glucomannan through a Maillard reaction led to gluten–glucomannan conjugates exhibiting increased β-sheet formation, decreased α-helix and β-turn motifs, and unfolded, loosened tertiary structures as the reaction occurred [20]. Although the products of gluten and saccharide glycation have modified physicochemical characteristics, little is known about the allergenicity of these conjugates. 

A few studies provide evidence that gluten protein glycosylation and glycation can reduce their allergenicity. *N*-glycosylation has been detected in high-molecular-weight glutenin [21], low-molecular-weight glutenin [22], and gliadin [23]. Song et al. [23] found that gliadin deglycosylation reduced its IgE-binding capacity and suggested that its *N*-glycans act as carbohydrate epitopes in wheat allergy. In the case of glycation, Wang et al. [24] observed that, when methylglyoxal (MGO), an intermediate Maillard reaction, reacted with glutenin, the MGO–glutenin conjugate formed resulted in a lower immune response than native glutenin, attributed to the induction of regulatory T cell (Treg) differentiation [24].

#### 2.1.2. Soybean Proteins

Soybean protein glycation through Maillard reactions can occur with fructose and fructooligosaccharides [25], lactose [26], and glucose [27,28], potentially reducing their allergenicity. For example, β-conglycinin antigenicity is reduced ~38% after 84 h of glycosylation with lactose [26], while it first decreased and then increased after 48 h of treatment with glucose [26]. This opposing trend is hypothesized to be an effect of increased advanced glycation end product (AGE) production after prolonged Maillard reactions [29]. It is therefore important to control Maillard-induced glycation up until the point after which protein allergenicity might increase due to AGEs.

#### 2.1.3. Faba Bean Proteins

In [30], the non-enzymatic glycation between a faba bean protein amino acid ε-amino group and the carbonyl group of maltodextrin occurred through Maillard conjugation [30]. The resulting faba bean protein–maltodextrin conjugate induced changes in the functional properties of the former component. In another study, the Maillard reaction between a faba bean protein isolate and *Leuconostoc pseudomesenteroides* DSM 20193- and *Weissella cibaria* Sj 1b-synthesized dextrans produced conjugates with improved functional properties, especially a high gel-strengthening ability [31]. However, no existing study has examined the effect of glycation on faba bean protein allergenicity. It can be assumed that, like soybean proteins, controlling the protein–sugar conjugation occurring during a Maillard reaction is necessary to avoid an increase in protein antigen allergenicity due to AGE production.

#### 2.1.4. Peanut Proteins

Peanut allergens can also be glycated to hide or destroy epitopes. For example, in [32], heating purified Ara h 2 and 6 together with glucose led to the destruction of their secondary structures and significantly reduced their allergenicity. The cause of the significant decrease in Ara h 2 IgE-binding capacity after glycation can be hypothesized as the masking of core antigenic epitopes upon the formation of a larger glycoprotein, blocking the antigen–antibody reaction [33,34]. The same trend was observed for Ara h 1 when heated for 20 min at high temperatures in the presence of glucose, whereby the glucosylated Ara h 1 had a 9000-fold-decreased IgE-binding capacity [32]. Peanut allergen glycation has been shown to be effective in significantly reducing their IgE-binding capacity and, subsequently, allergenicity.

### 2.2. Polyphenol Complexation

**Figure 3:**
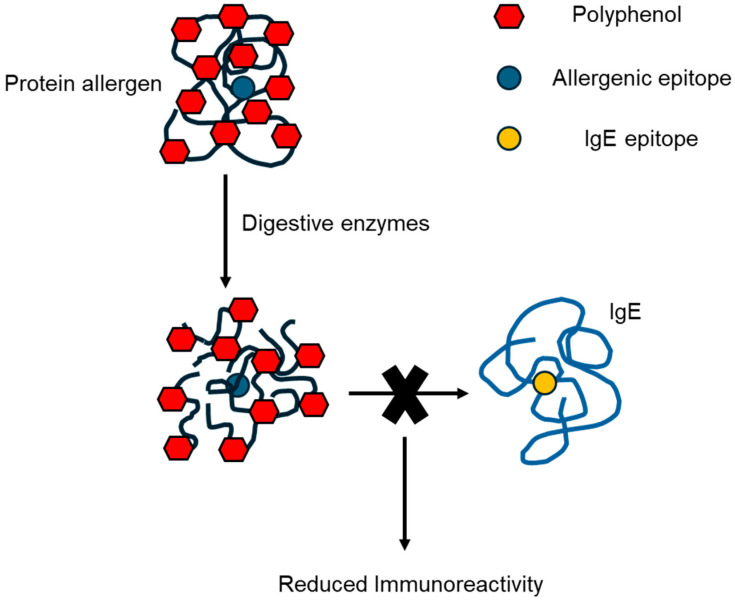
Possible mechanism for allergenicity reduction by polyphenol complexation. The polyphenols shield the allergenic epitope and/or the IgE epitope, resulting in less protein allergen immunoreactivity.

Polyphenols such as (-)-epigallocatechin gallate (EGCG) can potentially be bound to proteins to change their structure and allergenicity by hiding the allergen epitope inside the protein or covering it [35]. In addition, polyphenols can directly shield a linear IgE epitope, reducing its IgE-binding ability [35] (Figure 3). Another potential mechanism of allergenicity reduction is the formation of protein–polyphenol colloidal aggregate particles [36], a complexation that renders the protein allergen less immunoreactive when passing through the digestive tract.

#### 2.2.1. Gluten Proteins

Tannins have been shown to have gluten protein-binding abilities, resulting in reduced gluten sensitivity and allergy, especially gliadin [37]. Pérot et al. [38] reported that cranberry extract, which is rich in condensed tannins (up to 38 mg/g), inhibited the IgE response to wheat gliadin proteins, likely due to the change in protein conformation or the masking of the epitopes responsible for adverse reactions when polyphenol–gluten protein complexes were formed, similar to polyphenol–peanut protein complexes [39]. In a different study, EGCG was able to increase the relative helicity of α2-gliadin [40], one of the proteins implicated in celiac disease, potentially reducing the inflammatory response toward this protein [41]. Green tea extract also contains EGCG, which can react with partially digested gliadins, inhibiting the release of interleukins IL-6 and IL-8 in Caco-2 cells, which often serve as a model of the small intestinal epithelium, and reducing the effects of gliadin-stimulated monolayer permeability [41]. *In vivo* studies are needed to confirm the robustness of polyphenol reactions as a pretreatment reducing or eliminating the immune response in wheat allergy and/or celiac disease.

#### 2.2.2. Soybean Proteins

Lin et al. [42] covalently linked the soybean 7S protein fraction (β-conglycinin) with chlorogenic acid (CHA) and EGCG, finding that polyphenol conjugation resulted in protein structure changes, which facilitated the formation of cross-linked protein polymers and masked or destroyed the epitopes responsible for allergenic reactions [42]. The polyphenol-treated β-conglycinin fraction also exhibited reduced IgE-binding activity, histamine release, and anaphylactic shock symptoms *in vivo* [42]. The chemical modification of β-conglycinin also enhanced its antioxidant, emulsion, foaming, and foam stability properties, potentially through protein aggregation [42]. Besides affecting soybean protein allergenicity, particularly for β-conglycinin, polyphenol conjugation can also change their physical and chemical properties, possibly due to the modification of the modular structure of the proteins and, hence, their aggregate-forming ability.

#### 2.2.3. Faba Bean Proteins

According to [43], faba bean proteins and tannins can bind to form soluble complexes, which are then changed into insoluble precipitates by cross-linking. In the above study, when a high phenolic compound-to-protein ratio was present (0.04–0.37 mg catechin equivalents/mg of protein), there were strong faba bean protein–tannin interactions that resulted in insoluble complexes. The formation of aggregation complexes can suppress allergic sensitization, similarly to peanut protein–polyphenol complexes [36]. This study suggests that there is significant potential in using polyphenol–protein conjugates to reduce faba bean protein allergenicity and that more research should be conducted on this topic.

#### 2.2.4. Peanut Proteins

Chung et al. [44] reacted peanut proteins with a phenolic acid—caffeic acid—by oxidizing the tyrosine in the former and cross-linking it with the latter, reducing the allergenicity of the two major peanut allergens. Another study investigated the effects of covalent conjugation of Ara h 1 with EGCG and CHA on Ara h 1 allergenicity through the alkali method [45]. Enzyme-linked immunosorbent assay (ELISA) tests showed that the covalent conjugation of Ara h 1 with dietary polyphenols significantly reduced the Ara h 1 IgE-binding capacity and basophil histamine release. These effects could be attributed to protein conformation changes, wherein the Ara h 1 IgE epitope-binding site might be blocked by the polyphenol complex [45]. In a different study, the denaturation temperature of Ara h 1 also decreased, while its antioxidant activity increased upon covalent polyphenol conjugation. The allergenicity reduction efficiency is dependent on the allergen type because of the different epitope locations in various food matrices and allergens [46]. In addition, some polyphenols, when covalently bound to specific protein allergens, elicit a much lower allergenicity than when others are used [46].

In terms of covalent bonding, Ref. [35] showed that EGCG preferentially binds to the basic amino acids lysine and arginine, which can be used to reduce the overall protein allergenicity; however, basic amino acids can also undergo glycation. Also, glycation may enhance protein allergenicity via Maillard reactions [47]. A balance between glycation and polyphenol-binding ability is necessary to treat protein allergens. The covalent modification of proteins by polyphenols provides a new method of reducing the allergenicity of plant-based food allergens.

Chemical processes, such as glycation/glycosylation and polyphenol complexation, that result in protein structure and conformation changes generally lead to a reduction in protein allergenicity. This is due to the allergenic epitopes being hidden, masked, or destroyed upon allergen digestion; however, it is also possible for allergenicity to increase with glycation due to AGE formation. Because of this, polyphenol complexation is seemingly an easier-to-control and safer approach than Maillard reactions.

## 3. Biochemical Processes to Reduce Food Allergenicity

### 3.1. Fermentation through Microbial Activity

Fermentation is a process that can reduce allergenicity by protein degradation, acid-induced denaturation, glycosylation, and microbial activity-driven Maillard reactions [48]. From a more biological perspective, microbial strains and fermentation metabolites can activate Treg induction, regulate T-helper cell Th1/Th2 responses, maintain the epithelial barrier integrity, and increase gut microflora diversity [49], all mechanisms that contribute to reduction in allergenic reaction. Treg induction, for example, inhibits autoimmune responses and increases the threshold value for an immune response. In terms of Th1 and Th2 responses, allergies have been shown to favor the latter and be negatively regulated by Th1 cells. Changes in protein size (resulting from protein fragmentation) caused by fermentation normally maintain or contribute to a decrease in IgE-binding capacity in 2S albumins, legumins, and vicilins [50]. Fermentation is increasingly being “rediscovered” as a process due to its myriad nutritional and health effects, notably including food protein allergen degradation or transformation. 

#### 3.1.1. Gluten Proteins

Many lactic acid bacteria (LAB) that can metabolize gluten in the gut [51] could potentially be used for fermentation to reduce gluten allergenicity. Caminero et al. [51] characterized glutenasic human gut bacteria, predominantly isolating *Lactobacillus* (20% of the isolated bacteria). In other studies, gliadin proteolysis was also observed when *Lactobacillus* strains were present in sourdough [52,53]. In a study involving different LAB [54], a strain of *Lactococcus lactis*—LLGKC18—was found to cause degradation of the main allergenic proteins of gluten. After gluten fermentation by *Lactococcus lactis* LLGKC18, a significant decrease in the gliadin and glutenin content, allergenicity, and antigenicity was observed. It is possible that *Lactococcus lactis* LLGKC18 gluten fermentation can be used in gluten processing to hydrolyze the epitopes responsible for wheat allergy [54]. LAB are generally considered safe, encouraging their further application in fermentation.

Aside from *Lactobacillus*, the genera *Bifidobacterium* are also involved in gluten metabolism in the human gut [51], with some species, such as *B. longum*, *B. animalis*, and *B. bifidum*, exhibiting gliadin hydrolytic activity [55]. In fact, *B. longum* is commercially available in food as a probiotic for people with celiac disease [51]. Fermented gluten can also be used as a functional food which physico-nutritional properties can be controlled. Upon fermentation by *Bacillus subtilis*, for example, a tight 3D network gluten structure is formed after 168 h of fermentation; after which, the microstructure becomes dense, potentially due to proteolysis [56]. A particulate, dense microstructure results in a highly viscous network [57]. Fermented gluten also exhibits higher free radical-scavenging activity and iron reduction capacity, suggesting that it could be developed as a food product to alleviate oxidative stress [56].

#### 3.1.2. Soybean Proteins

Many soybean products have been tested for allergenicity after fermentation [58,59,60,61]. In a study by [58], the IgE-binding capacity of a soy protein isolate was reduced by 83.8%–94.8% after *Lactobacillus plantarum* fermentation, potentially due to alterations in the primary and higher protein structures [58]. In a different study using *Bacillus subtilis* KCCM11438P as a fermentation strain in soybean meal, β-conglycinin and glycinin were found to be degraded and, after 24 h of solid-state fermentation, reduced by 70% and 42%, respectively [62]. In [60], *Enterococcus faecalis*-fermented soymilk exhibited the complete degradation of the β and acidic subunits of β-conglycinin and glycinin, respectively, as observed through SDS-PAGE. Meanwhile, in another study, the fermentation of basic glycinin subunits was not as effective as that of its acidic ones, probably due to the presence of more hydrophobic amino acids, forming a hydrolysis-resistant hydrophobic core [61].

The degree of degradation or reduction in protein allergenicity is dependent on the strain type and fermentation time. For instance, the α subunit of β-conglycinin was reduced to 0.73 µg/mg after 72 h of *Saccharomyces cerevisiae* fermentation, registering the biggest reduction in comparison to *Bacillus subtilis*, *Geotrichum candidum*, *Endomycopsis fibuligera*, and *Candida utilis* [59]. Similar results have been shown for the profilin allergen Gly m 3 content after the fermentation of soybean meal, paste, and yogurt using strains such as *Bifidobacterium lactic*, *Lactobacillus plantarum*, and *Saccharomyces cerevisiae* [63].

#### 3.1.3. Faba Bean Proteins

In [64], the lactic acid fermentation of faba bean flour was performed to boost its nutritional value, with the results suggesting that it induced faba bean protein hydrolysis, causing changes in its functional properties. Fermentation can improve protein digestibility; lower the glycemic index; and destroy antinutritional factors such as faba bean-associated trypsin inhibitors, phytic acid, saponins, and galactosides [65]. According to a study by Chandra-Hioe et al. [66], faba bean flour fermentation effectively boosted in vitro digestibility while lowering trypsin inhibitor activity. Nevertheless, no study on reducing faba bean allergenicity by fermentation was found in the literature.

#### 3.1.4. Peanut Proteins

In [67], it was found that Ara h 1 and 2 allergenicity was significantly reduced in *Bacillus subtilis*-fermented peanuts. The possible mechanisms reducing allergenicity include the following processes, all enacted by components produced during fermentation: protein allergen hydrolysis by proteases, organic acids affecting peanut protein allergenicity, and free radicals generated through glycosylation and Maillard reactions forming glycoproteins and lipoproteins from the peanut allergens. It is also possible that the active components produced by the microorganisms during fermentation may help the body balance the Th1/Th2 immune responses [68].

Another Bacillus species, *Bacillus natto*, has been used in the fermentation of raw peanut pulp [48]. *Bacillus natto* is a safe, acid- and heat-resistant probiotic [48] that secretes an enzyme more efficient in degrading proteins than *Bacillus subtilis* [48]. In the study, after 60 h of *Bacillus natto* fermentation, the IgE-binding of raw peanut pulp was reduced by around 77% compared to 67% using autoclaving alone [48]. When *Bacillus natto* was combined with *Lactobacillus plantarum* in mixed-strain fermentation, the IgE-binding capacity of raw crushed peanuts was reduced by 90.2% due to epitope masking and destruction [48].

Although fermentation can reduce peanut protein allergenicity, it can also affect the physico-sensorial properties of peanut products. For example, the fermentation of peanut milk using a mixed culture of *Lactobacillus delbrueckii* subsp. *bulgaricus* and *Streptococcus thermophilus* was shown to decrease its beany flavor and increase its whiteness, viscosity, gumminess, and smoothness [69].

### 3.2. Enzymatic Catalysis

Enzymatic catalysis can be a type of enzymatic degradation and/or cross-linking. Enzymatic degradation or proteolysis (catalyzed hydrolysis) mainly destroys the IgE-binding components to reduce allergenicity [17]. However, protein resistance to enzymatic digestion, such as pepsin digestion, cannot be used as a good predictor of allergenicity. Lipids also play a role during the digestion process, as, through their interactions with the allergenic proteins, they may stabilize them and preserve their structure-related allergenicity [50]. Meanwhile, enzymatic cross-linking is a non-thermal process that produces high-molecular-weight polymerized allergens with reduced immunoreactivity and IgE-binding potential [70]. Both processes can be combined to substantially reduce food protein allergenicity.

#### 3.2.1. Gluten Proteins

Previous studies using enzymatic cross-linking are included below. In some studies, wheat proteins were treated with transglutaminase to develop hypoallergenic flour for celiac patients [71,72], whereby the allergenic epitopes were modified through glutamine residues, resulting in a reduction in toxicity for these patients [71,73,74]. The same observation was obtained for gliadin immunoreactivity following transglutaminase treatment [75]. However, the transglutaminase enzyme was also shown to deamidate glutamine residues within gliadin fragments into glutamic acid, increasing the binding affinity of gliadin peptides to HLA-DQ2/DQ8—the proteins associated with a greater disposition to celiac disease [76]. In a different study that took advantage of both enzymatic cross-linking and digestion, Ref. [77] used single- and two-step enzymatic treatments to modify whey protein immunoreactivity through hydrolysis by the peptidases and prolyl endopeptidase of high-molecular-weight ω-gliadins and glutenins. After proteolysis, the wheat allergens were cross-linked through transglutaminase. This two-step enzymatic modification resulted in gliadin and glutenin reduction. Meanwhile, limited chymotrypsin digestion followed by microbial transglutaminase treatment led to a substantial reduction in allergenicity and enhancement in the emulsifying activity of the hydrolyzed gluten [78]. The transglutaminase treatment also increased the solubility of the wheat gluten hydrolysate [78].

Another group of proteases that can degrade gluten proteins comprises proline-specific peptidases [79]. In the study by [79], prolyl endopeptidase from *Aspergillus niger* was used to degrade gluten in wheat bran and bread drink. An engineered protease, Kuma030, reported by Wolf et al. [80], was found to recognize the tripeptide sequences in the immunogenic regions of gliadin. This treatment destroyed the ability of gliadin to stimulate a T cell response. The engineered enzyme is a potential oral therapeutic for celiac patients [80].

#### 3.2.2. Soybean Proteins

Enzymatic cross-linking by microbial transglutaminase (MTGase) has been shown to reduce soymilk antigenicity to 67.8% due to the formation of protein polymers [81]. In addition, treatment with MTGase has been shown to reduce tofu allergenicity to a certain extent, possibly due to the structural changes in the cross-linked tofu [82]. Meanwhile, digestion with alcalase, trypsin, and pepsin reduces soybean protein allergenicity [63,83]. In [83], it was noted by sodium dodecyl sulfate-polyacrylamide gel electrophoresis (SDS-PAGE) that the α’, α, and β subunits of β-conglycinin disappeared, while both the basic and acidic subunits of glycinin were degraded, after alcalase treatment [83]. On the other hand, treatment with trypsin seemed to induce minimal degradation in both β-conglycinin and glycinin [83]. In other studies, the allergenicity of glycinin and the soybean 2S protein was also tested after a combined treatment of pepsin and chymotrypsin [84,85]: Lee et al. [84] found reduced overall glycinin allergenicity, while the allergenicity of the soybean 2S protein was partially reduced after peptic digestion and slightly increased following combined chymotryptic and peptic hydrolysis [85].

#### 3.2.3. Faba Bean Proteins

In a study using enzymatic cross-linking, Liu et al. [86] reported that MTGase can alter the physicochemical properties of faba bean proteins by inducing cross-linking and increasing the net charge on the protein. In another study where enzymatic cross-linking was also performed, faba bean proteins were treated with transglutaminase, which increased their colloidal stability due to improved electrostatic stability and intra-particle cross-linking [87]. However, no effect on allergenicity has been reported thus far, leaving a great research gap. Proteolysis of faba bean proteins at a neutral pH using pepsin, trypsin, flavourzyme, alcalase, or neutrase significantly increased protein solubility, foaming, and oil-holding capacities [88,89], with no tested effects on allergenicity—another topic requiring further research.

#### 3.2.4. Peanut Proteins

The enzymatic cross-linking of peanut allergens can be accomplished with the microbial enzyme MTGase. In one study, MTGase was used to catalyze a cross-linking reaction of a recombinant form of Ara h 1 (rAra h 1) [90], where ε-(γ-glutamyl) lysine isopeptide bonds were formed, which resulted in a relatively stable structure. The cross-linking process also significantly reduced rAra h 1 immunoreactivity by roughly 30%, potentially through linear epitope exposure due to a decrease in the content of conserved secondary structures. A reduction in the rAra h 1 surface hydrophobic index after MTGase-catalyzed cross-linking and increased steric hindrance also made it more difficult to bind with antibodies, possibly preventing subsequent allergic reactions [90].

The enzymatic degradation of some peanut allergens can be performed using pepsin. Its use is not recommended for Ara h 2 and 6 because of their stability towards this form of digestion [91] and their hydrolysis resistance, aided by the presence of strong disulfide bridges stabilizing the secondary or tertiary structure of some protein allergens [91]. Other enzymes can be used to reduce peanut allergen IgE reactivity. Cabanillas et al. [92] observed that, when roasted peanuts were treated with alcalase for 30 min, the IgE binding of Ara h 1, 2, and 3 was significantly reduced. The reverse effect was reported for the flavourzyme treatment, and when alcalase was sequentially combined with the flavourzyme, the IgE reactivity of the peanut samples was reduced by 100% [92]. Despite its promising effectiveness, some of the limitations of enzymatic catalysis remain. For instance, it is possible that, upon enzymatic degradation, partially hydrolyzed fragments may still be highly immunoreactive [84], interior hidden epitopes may be exposed [85], and new epitopes may form [93]. In addition, enzymatic catalysis has specific requirements for different allergens and necessary treatment conditions (such as pH, temperature, hydrolysis time, and enzyme amount) [47,94]. It might also result in flavor profile changes, limiting its application in food development.

Similarly to chemical treatments, biochemical treatments such as fermentation and enzymatic catalysis can result in protein structure and conformation changes, rendering a protein less allergenic. Among the chemical and biochemical processes discussed, fermentation offers the more effective way of reducing allergenicity, as microbial action combines protein degradation, denaturation, glycosylation, and Maillard reactions in one treatment. Besides reducing the allergenicity of plant protein ingredients, fermentation modifies the physical, sensory, and nutritional properties of the food matrix, thereby allowing new hypoallergenic products with distinct characteristics to be developed. 

## 4. Emerging Technologies to Increase the Effectiveness of Chemical and Biochemical Treatments

### 4.1. Physical Treatments

Microwave, high-pressure, and ultrasonication processes are some of the potential physical pretreatments that might be combined with chemical and biochemical techniques to reduce food allergenicity. Microwave energy may reduce allergenicity through (1) protein aggregation and structural changes, (2) the destruction of allergen epitopes through subsequent enzymatic hydrolysis, and (3) the disruption of disulfide links in the protein, lowering their stability and increasing their susceptibility to the enzyme breakdown [95]. This was the case for a microwave treatment, reducing gliadin allergenicity by ~99% in the ELISA test [96]. On the other hand, the microwave treatment of gluten proteins can also render gluten with increased immunotoxicity for people with celiac disease due to increased gluten digestibility [97]. For soybean cupins, β-conglycinin, and glycinin, the allergenicity was reduced to 24.7% after microwaving, despite preserving the cupin structures [98]. By analyzing protein digestibility, peanut cupin allergenicity can be deduced. Vanga et al. [99] observed that peanut cupin allergenicity increased after a microwave treatment (the protein digestibility was ~90%). Moreover, when combined with polyphenol cross-linking, microwaving at 500 watts (W) for 1–3 min resulted in an average 9.7% reduction in the allergenicity of legumes and nuts in a meta-analysis study [100].

Another technique—high hydrostatic pressure—may inactivate certain conformational epitopes and can be combined with enzymatic hydrolysis to interfere with linear epitopes [101]. In the same meta-analysis study mentioned above, an average 70.5% reduction in the allergenicity of legumes and nuts was obtained using a combination of high hydrostatic pressure at 100–600 megapascals (Mpa) at 50 °C for 15 min followed by hydrolysis at 50 °C for 15 min [100].

High-intensity ultrasound treatment could modify the protein conformation by affecting noncovalent interactions and disrupting the quaternary and/or tertiary structures of globular proteins to expose more hydrolysis sites, which could then be acted upon by proteases during an enzymatic treatment [102]. Soy proteins and allergens are generally resistant to enzymes, but when enzymatic digestion is combined with ultrasound treatment, soy proteins can be easily hydrolyzed [103]. Pretreatment is a necessary step to alter the structural characteristics of soy proteins by changing their conformation or exposing previously hidden sites to facilitate enzymatic action or degradation. An overview of the different chemical, biochemical, and physical treatments discussed in this review and the degree of allergenicity reduction in nut and legume allergens is presented in Table 1. From these data, it is worth noting that physical processes such as microwave, high pressure, and ultrasound, when used alone, without successive degradation or conjugation reactions to destroy the conformation or antibody-binding ability of the protein epitopes, may sometimes result in increased allergenicity, depending on the protein allergen. It is suggested to combine these processes with subsequent chemical and biochemical treatments.

### 4.2. Colloidal Complexation to Bind Allergens

**Figure 4:**
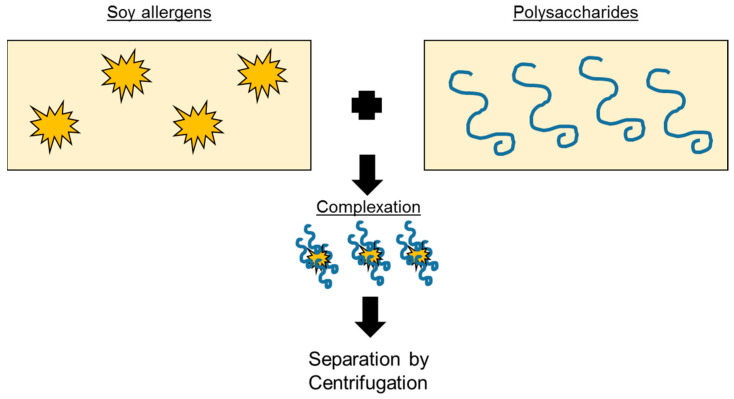
Scheme for a reduction in the protein allergen content (for example, in soymilk) by colloidal complexation with polysaccharides.

Depending on the pH, proteins can be positively or negatively charged, while polysaccharides can carry negative charges at certain pH ranges. These electrical charges on the protein or polysaccharide may lead to electrostatic interactions [104]. In addition to these interactions, hydrogen bonding and hydrophobic interactions can also stabilize protein–polysaccharide aggregates. Anionic polysaccharides such as pectin (with an isoelectric point, pI, of 3.5) can form complex coacervates with proteins that possess positive charges at 3.5 < pH < pI of protein through electrostatic interactions [104]. These protein–polysaccharide complexes can then be separated from the rest of the liquid medium via the subsequent processing steps—for example, centrifugation (Figure 4).

In the food industry, positively charged globular proteins are complexed with ionic polysaccharides. Such a complexation can be a novel strategy to bind protein allergens in liquid foods such as soymilk. For example, soy protein isolates (SPIs) have been shown to form stable coacervates with cationic polysaccharide chitosan or carboxymethyl cellulose after heating at specific pH ranges [105]. It is also possible to combine glycation through a Maillard reaction and the electrostatic formation of coacervates. Huang et al. [106] studied the occurrence of glycation with maltose during coacervation at different temperatures for 12 h. They found that increasing the Maillard reaction resulted in the amino groups (initially positively charged) of chitosan reacting with maltose, thereby producing a negative charge. These electric charge changes led to decreased aggregation and higher stability [106]. 

Another way to form protein–polysaccharide complexes is by enzymatic cross-linking. For example, SPI has been shown to form double-network gels with sugar beet pectin during thermal treatment and laccase catalysis [107]. These gels can stabilize emulsions, and potentially, the separation of protein allergens in SPI can be achieved after further processing. So far, no study has been carried out to test the efficiency and effectiveness of using protein–polysaccharide colloidal systems to bind protein allergens and reduce their levels in liquid foods. It remains to be seen whether this proposed strategy can be adopted widely in the production of hypoallergenic soymilk formula.

## 5. Challenges and Outlook

Food allergens pose one of the major hurdles when it comes to the widespread adoption of plant-based foods and meat analogs. There are many avenues that remain to be developed and refined in terms of the chemical and biochemical modification of the protein allergens from gluten and leguminous foods. Polyphenol treatment is a promising research area, mainly because there are many polyphenol families that can be used to modify allergens, as well as potentially undiscovered compounds from other food sources. Enzymatic modification is also exciting, since there are many still-undiscovered enzymes requiring biochemical characterization. Fermentation has largely been relegated to the production of new sensory compounds and unique textures, but when coupled with the discovery of allergen-metabolizing bacteria in the human gut microbiome, it is gearing towards finding the main causes and treatments of food allergies. This raises the question of what these processes change or modify at the molecular level of proteins and amino acids. Trying to answer this question makes protein modification more targeted and specific, without losing the nutritional value or sensory–physical properties of the food matrix. One of the strategies proposed is protein–polysaccharide complexation to bind protein allergens and separate them from the liquid medium. The efficiency and effectiveness of this technique have not been studied yet, and it remains to be seen whether it is possible to use it to reduce the protein allergen content in soymilk, as an example. Among the techniques mentioned in this review, fermentation, on its own, appears to be the most effective means of reducing allergenicity. Other processes, such as glycation/glycosylation, polyphenol complexation, and enzymatic catalysis, have a lower effectiveness when used on their own. For these treatments, it is advisable to combine them with physical methods such as the use of microwaves, high pressure, and ultrasound. Newer technologies such as cold plasma treatment and pulsed electric field should also be considered as treatments for allergenicity reduction. The development of hypoallergenic food products will soon be one of the major advances in food processing and technology.

## Figures and Tables

**Table 1:** Different chemical, biochemical, and physical processing techniques to reduce the allergenicity of plant-based food allergens ^a^.

Processing	Measured Sample Form	Processing Condition	n ^b^	Actual Allergenicity Reduction (%) ^c^		
				Min	Max	Ave
Maillard reaction *	Protein	Reaction at 40–100 °C, 20 min–5 h	4 (40)	0	91.4	17.53
Maillard reaction *	Protein	Reaction at 60 °C, 24–72 h	1 (20)	11.12	54.23	26.82
Maillard reaction *	Whole sample	Reaction at 170 °C, 20 min	2 (20)	16.89	49.16	37.98
Cross-linking with polyphenol *	Protein	Reaction at 25–100 °C, 1–24 h	3 (49)	0	82.07	34.06
Proteolytic hydrolysis ^†^	Protein	Temperature 37–60 °C, 15 min–8 h	7 (70)	0	100	55.35
Proteolytic hydrolysis ^†^	Whole sample	Temperature 45–55 °C, 1–2 h	3 (30)	19.27	99.95	75.43
Fermentation ^†^	Protein	Temperature 33–37 °C, 24–48 h	3 (25)	0	100	75.29
Fermentation ^†^	Whole sample	Temperature 30–37 °C, 24–48 h	4 (47)	15.16	99.15	66.74
High hydrostatic pressure (HHP) and proteolytic hydrolysis ^‡^	Protein	HHP at 100–600 MPa, 50 °C for 15 min, then hydrolysis at 50 °C for 15 min	1 (24)	32.11	99.89	70.49
Microwaving and cross-linking with polyphenol ^‡^	Whole sample	Microwaving at 500 W for 1–3 min	1 (8)	0	23.96	9.69
Maillard reaction and cross-linking with polyphenol ^‡^	Whole sample	Roasting at 170 °C for 20 min, Sugar: sucrose or glucose	1 (16)	0	40.37	28.06
Fermentation and proteolytic hydrolysis ^‡^	Whole sample	Fermentation at 30–37 °C for 24 h, then hydrolysis at 50 °C for 15 min	1 (10)	77.89	96.39	85.93

^a^ Adapted from [100]. ^b^ *n* = number of studies (number of data extracted and entered into RevMan 5.4 software, according to [100]). Since one of the studies used one or more different processing conditions to investigate its effect on protein allergenicity, one study could generate more than one set of data. ^c^ Actual allergenicity reduction obtained from raw data. * Chemical process. ^†^ Enzymatic process. ^‡^ Combined processes.

## Data Availability

No new data were created or analyzed in this study. Data sharing is not applicable to this article.
